# Overexpression of Interleukin-23 and Interleukin-17 in the Lesion of Pemphigus Vulgaris: A Preliminary Study

**DOI:** 10.1155/2014/463928

**Published:** 2014-05-11

**Authors:** Jixin Xue, Wenting Su, Zhiwei Chen, Youhui Ke, Xiaojing Du, Qiaochu Zhou

**Affiliations:** ^1^Department of Hand and Plastic Surgery, The 2nd Affiliated Hospital & Yuying Children's Hospital of Wenzhou Medical University, No. 109, Xueyuan West Road, Wenzhou, Zhejiang 325027, China; ^2^Department of Dermatology, Wenzhou Hospital of Integrated Traditional Chinese and Western Medicine, No. 75, Jinxiu Road, Wenzhou, Zhejiang 325027, China

## Abstract

IL-23/IL-17 axis has been identified as major factor involved in the pathogenesis of several autoimmune diseases; yet its pathogenetic role in pemphigus vulgaris (PV) remains controversial. The aim of this research was to investigate the potential role of IL-23/IL-17 axis in the immunopathogenesis of PV, and correlation between IL-23+ cells and IL-17+ cells was also evaluated. For this purpose, ten patients with PV, three patients with pemphigus foliaceus (PF), and six healthy individuals were allocated to this research. The lesional skin biopsy specimens were obtained before treatment. Then immunofluorescence staining was performed to analyze the expression of IL-23 and IL-17 in the PV/PF patients and the healthy individuals. The results showed that the numbers of IL-23+ and IL-17+ cells were significantly higher in PV lesions, compared to PF lesions and normal control skins, respectively (all *P* < 0.05). Moreover, the correlation between IL-23+ cells and IL-17+ cells was significant (*r* = 0.7546; *P* < 0.05). Taken together, our results provided evidence that the IL-23/IL-17 axis may play a crucial role in the immunopathogenesis of PV and may serve as novel therapeutic target for PV.

## 1. Introduction

Pemphigus vulgaris (PV), the most prevalent type of pemphigus, is a life-threatening autoimmune bullous disease characterized by an autoantibody predominant target epitopes to desmoglein 3 (Dsg3), a desmosomal cell adhesion glycoprotein [1–4]. It can be considered as a chronic organ-specific disorder because the autoimmune injury which leads to the formation of intraepidermal blisters and acantholysis is confined to the skin and mucosa. A number of studies about PV, using patient samples or animal models, have been previously reported. However, despite recent advancements, the pathogenesis of PV to date remains to be fully elucidated.

Autoreactive T cells are thought to play a central role in the pathogenesis of PV [1, 5–7]. Recently, emerging findings put the spotlight on the contribution of a newly discovered subset of Interleukin-17 (IL-17) producing T helper (Th) cells accordingly named Th17 cells to autoimmune states [8–12]. Furthermore, over the past few years, increasing evidence suggested that the development and maintenance of Th17 cells have been linked to Interleukin-23 (IL-23), a key initiating cytokine in the development of autoimmunity [10]. It was reported that IL-23 was mainly secreted by macrophages and dendritic cells (DCs) and IL-23 promoted the expansion of the novel Th17 population [13–15]. As a result, a crucial role was proposed for the IL-23/IL-17 axis in mediating tissue inflammation and autoimmunity recently, such as psoriasis [16, 17]. However, despite the current evidences indicating that IL-17 may play an important role in PV [18, 19], few reports have explored the crucial role of the IL-23/IL-17 axis in the immunopathogenesis of PV.

We thus hypothesized that the IL-23/IL-17 axis will also be functionally involved in the development and maintenance of PV. In this study, we examined the immunoexpression of IL-23 and IL-17 in the lesional biopsy specimens from 10 cases of PV, comparing the results with those of PF patients and normal control skins from 6 healthy individuals, and evaluated the correlation between IL-23+ cells and IL-17+ cells; moreover, the sources of IL-23 were also evaluated.

## 2. Materials and Methods

### 2.1. Subjects

In this descriptive-analytical study, the subjects were 10 (3 men and 7 women) unrelated patients with PV and 3 (1 man and 2 women) patients with pemphigus foliaceus (PF) diagnosed by clinical and immunohistochemical criteria. The lesional biopsy specimens of the patients were obtained before treatment during the active phase for the purpose of evaluating the acute state. Specimens were stored at −80°C until use. The normal control skins (eyelid skin) were taken from 6 (1 man and 5 women) healthy people who were selected randomly. This study was approved by our local Ethics Committee, and written informed consent was obtained from all participants.

### 2.2. Immunofluorescence Staining

Immunofluorescence staining of cryosections from those specimens, both the PV patients and the healthy individuals, was performed with the following primary antibodies: rabbit anti-human IL-17 pAb (Santa Cruz), goat anti-human IL-23 pAb (Santa Cruz), and mouse anti-human CD163 mAb (Santa Cruz). For negative control preparations, the first antibodies were replaced with mouse F(ab′)_2_ IgG (Abcam), an irrelevant isotype control. The second antibodies are Alexa Fluor 488 goat anti-mouse IgG (Invitrogen), Alexa Fluor 555 rabbit anti-goat IgG (Invitrogen), and Alexa Fluor 555 goat anti-rabbit IgG (Invitrogen). The counts of IL-17+ cells, possibly Th17 cells (IL-17), IL-23+ cells (IL-23), and CD163+ cells, possibly macrophages (CD163), in three sections were quantitatively evaluated.

### 2.3. Statistical Analysis

The counts of positive staining cells were initially reported in the form of descriptive statistics. Statistical analysis was performed with the GraphPad Prism-5 statistical package. Unpaired *t*-test was utilized for comparison of IL-17 and IL-23 between the three groups and the significance of the correlation was assessed by* Pearson* test. A *P* value < 0.05 was considered statistically significant in this research

## 3. Results

### 3.1. Expression of IL-23/IL-17 in PV Lesions and Normal Control Skin

**Figure 1:**
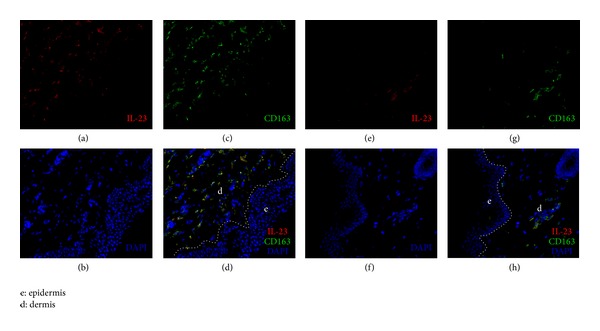
Immunofluorescence studies of specimens from lesions of PV (a–d) and from normal skin of control (e–h). The red and green stained cells were detected by antibodies to IL-23 and CD163, respectively.

**Figure 2:**
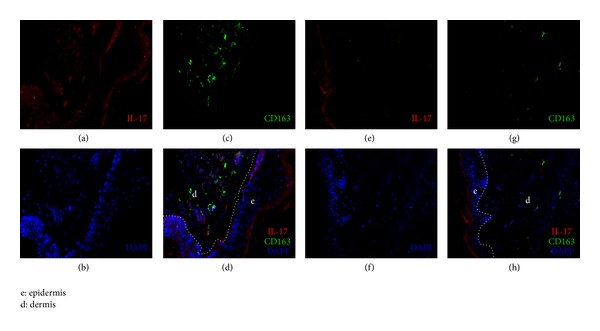
Immunofluorescence studies of lesional skin specimens (a–d) and control skin specimens (e–h). The red and green stained cells were detected by antibodies to IL-17 and CD163, respectively.

**Figure 3:**
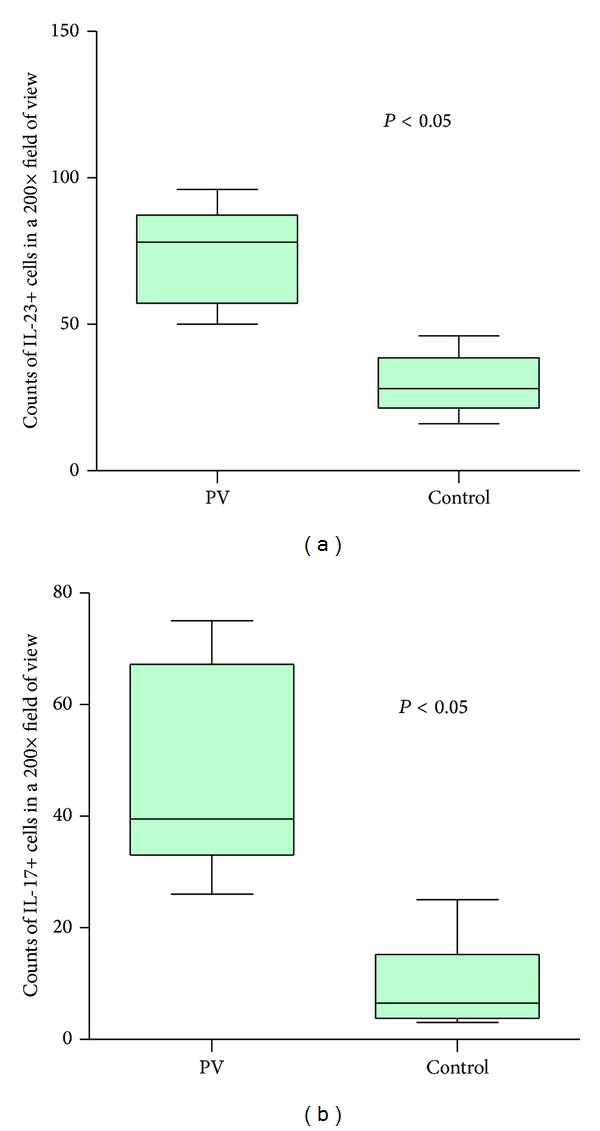
Quantification of the number of IL-23+ cells (a) and IL-17+ cells (b) in PV and normal control group. The significance of the differences was assessed by an unpaired *t*-test.

The numbers of IL-23+ cells and IL-17+ cells were significantly increased in PV lesions (Figures 1(a)–1(d) and Figures 2(a)–2(d)), compared to healthy controls (Figures 1(e)–1(h); Figures 2(e)–2(h); Figure 3, *P* < 0.05). The counts of IL-23+ cells in each 200x field of view in PV lesions and normal control skin were 73.70 ± 5.315 and 29.50 ± 4.448, respectively (Figure 3(a), *P* < 0.05), while the numbers of IL-17+ cells in each 200x field of view in PV lesions and normal control skin were 46.60 ± 5.673 and 9.50 ± 3.354, respectively (Figure 3(b), *P* < 0.05). Besides, the IL-23+ staining was overlapped with the CD163+ macrophages (Figures 1(d) and 1(h)), which indicated that the IL-23 was secreted by macrophages.

### 3.2. Correlations between IL-23+ Cells and IL-17+ Cells in PV Lesions

**Figure 4:**
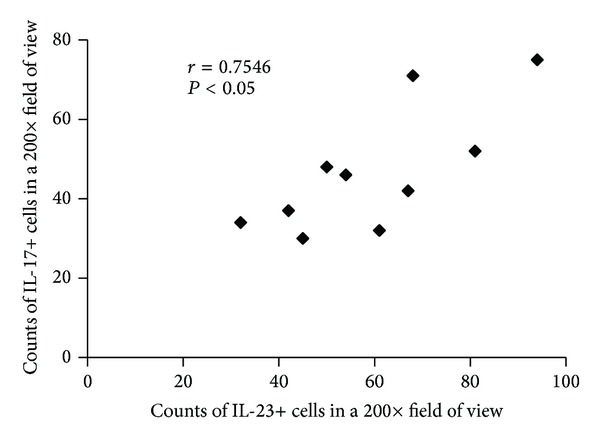
Correlation between IL-23+ cells and IL-17+ cells in the lesions of PV. The counts of IL-23+ cells and IL-17+ cells were the average number of three 200x fields of views from three sections. Significance of the correlation was assessed by Pearson test.

As the IL-23+ staining was overlapped with the CD163+ macrophages (Figures 1(d) and 1(h)), we adopted the number of CD163+ cells instead of IL-23+ cells' counts for the correlation analysis (Figure 2). Based on Pearson test, the correlation between IL-23+ cells and IL-17+ cells in PV lesions was significant (*r* = 0.7546; *P* < 0.05) (Figure 4).

### 3.3. Expression of IL-23/IL-17 in PF Lesions

The counts of IL-23+ cells and IL-17+ cells in each 200x field of view in PF lesions were 42.67 ± 5.812 and 21.33 ± 4.485, respectively (see Supplementary Figure in the Supplementary Material available online at http://dx.doi.org/10.1155/2014/463928), where we found a statistically significant decrease in number compared to PV (*P* < 0.05). But no statistically significant differences of these numbers between PF and the control group were identified.

## 4. Discussion

In the current research, we examined the lesions and found that both IL-23 and IL-17 were overexpressed in PV patients, compared to the healthy controls and PF patients. More importantly, the results showed a correlation between IL-23+ cells and IL-17+ cells. Finally, this study demonstrated that the IL-23 was secreted by a cell population in dermis expressing CD163, which was considered as a surface marker of the macrophages [20]. In a previous experimental study conducted by Arakawa et al., the authors presented the possibility of Th17 (IL-17+ cells) that played an important role in the pathogenesis of PV. They quantified Th17 cells in lesional biopsy specimens from PV patients and found a significantly higher expression of IL-17+ cells compared to controls [18], which was also confirmed by our results. However, the importance of IL-23 was not investigated in Arakawa's study.

To our knowledge, IL-23 was initially discovered in 2000 by Oppmann and colleagues [21]. IL-23 was clearly not required for the initial induction of IL-17 production in naive T cells either in vitro or in vivo. However, production of IL-17 by memory effector cells was clearly enhanced in the presence of IL-23 [21], and it was shown that IL-23 maintained expression of IL-17 in activated Th17 cells [22]. Since its discovery, IL-23/IL-17 axis has been linked to the pathogenesis of various autoimmunity disorders, such as psoriasis and systemic lupus erythematosus [16, 23–25]. However, a limited number of reports have explored the crucial role of the IL-23/IL-17 axis in the immunopathogenesis of PV. Taken together, this provided us with the basis for a rising interest in the IL-23/IL-17 axis in PV. Our results showed overexpression of IL-23 and IL-17 in the lesion of PV patients and a correlation between IL-23+ cells and IL-17+ cells, which suggested that the IL-23/IL-17 axis probably played an important role in the immunopathogenesis of PV. The first issue which should be considered is which type of immunopathology IL-23/IL-17 axis may show in PV. One possibility is that it may be the initiators of the disease. Alternatively, IL-23/IL-17 axis may not be a cause but a result of the disease; in other words, it may possibly appear in a protective response to maintain epithelial homoeostasis. Future studies are required in order to better explore this important issue.

Another important issue is the source of IL-23. Traditionally, IL-23 was found to be expressed mainly by macrophages and dendritic cells (DCs) in dermis [21, 26]. In an earlier study, CD163 was recommend to be considered as an alternative marker to identify dermal macrophages for its more specific and more useful in flow cytometry applications [20]. Our findings that the IL-23+ staining was overlapped with the CD163+ macrophages in all specimens indicated that the IL-23 in dermis was expressed mainly by the CD163+ macrophages. In other words, macrophages, which have been confirmed to play an important role in the pathogenesis of psoriasis [27], may be also involved in the pathogenesis of PV.

In summary, the present study suggested the importance of the IL-23/IL-17 axis in the development of PV, which provided us with some clues for the elucidation of the pathogenesis of PV. However, several problems in the current study remain to be resolved. The reason why more IL-17+ cells and IL-23+ cells were present in lesional specimens from PV than those from PF is not certain. Actually, the accurate role of IL-23/IL-17 axis in the pathogenesis of PV is still unresolved in the present study. Additionally, the current research had some limitations that should be noted, such as the following: (i) the results were descriptive mainly and lacked some mechanistic studies, (ii) as a retrospective study, the serum levels of IL-23 and IL-17 were not tested, and a correlation between IL-23/IL-17 and disease activity or antibody titers was not investigated, and (iii) the relatively small number of patients was employed in this study. Prospectively, further study of the IL-23/IL-17 pathway in the pathogenesis of PV in mice model may be encouraged to further validate our hypothesis. Additionally, we hypothesized that targeting the IL-23/Th17 pathway maybe a highly effective therapeutic approach in the treatment of PV. Future studies are required in order to better explore this pathway as a potential therapeutic target.

## 5. Conclusions

In conclusion, our present study provides evidence that the expression of IL-23 and IL-17 was elevated and correlated in PV patients, which suggested the crucial role of the IL-23/IL-17 axis in the development of PV and provided us with some clues for the elucidation of the pathogenesis of PV.

## Supplementary Material

The numbers of IL-23+ cells and IL-17+ cells were significantly decreased in PF lesions, while compared to PV. But no statistically significant differences of these nubmers between PF and the control group were identified.

## Abbreviations

PV: Pemphigus vulgaris
Dsg3: Desmoglein 3
DCs: Dendritic cells
IL-23: Interleukin-23
IL-17: Interleukin-17.
