# Supplementary material for: Overexpression of Interleukin-23 and Interleukin-17 in the Lesion of Pemphigus Vulgaris: A Preliminary Study
Source: Mediators Inflamm. 2014 May 11;2014:463928. doi: 10.1155/2014/463928 (PMC4037576; doi:10.1155/2014/463928)
Supplement: Supplementary file 1 — The numbers of IL-23+ cells and IL-17+ cells were significantly decreased in PF lesions, while compared to PV. But no statistically significant differences of these nubmers between PF and the control group were identified. [file 463928.f1.pdf]

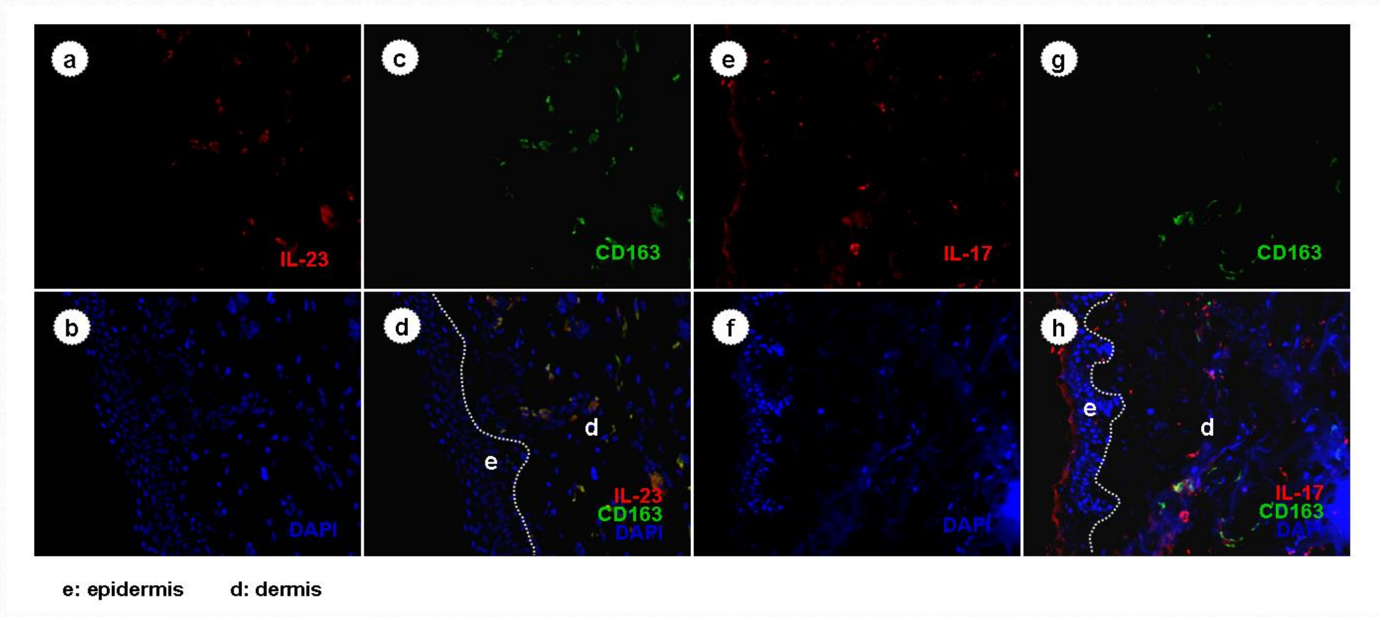


**Supplementary Fig.** Immunofluorescence studies of specimens from lesions of PF. Positively stained cells were detected by antibodies to IL-23 (red) and CD163 (green) (a-d), or by antibodies to IL-17 (red) and CD163 (green) (e-h).
